# Coding-Complete Genome Sequence of a SARS-CoV-2 Strain Isolated in Gilgit, Pakistan

**DOI:** 10.1128/MRA.01151-20

**Published:** 2021-04-01

**Authors:** Aneela Javed, Saifullah Khan Niazi, Eijaz Ghani, Maham Yamin, Muhammad Saqib, Hussnain A. Janjua, Ali Zohaib

**Affiliations:** a Atta-ur-Rahman School of Applied Biosciences, National University of Science and Technology, Islamabad, Pakistan; b Armed Forces Institute of Pathology (AFIP), Rawalpindi, Pakistan; c Faculty of Veterinary Science, University of Agriculture Faisalabad, Faisalabad, Pakistan; d Faculty of Veterinary and Animal Sciences, The Islamia University of Bahawalpur, Bahawalpur, Pakistan; DOE Joint Genome Institute

## Abstract

Here, we report the coding-complete genome sequence of a severe acute respiratory syndrome coronavirus 2 (SARS-CoV-2) isolate obtained from a nasopharyngeal swab from the first patient with COVID-19 in Gilgit, Pakistan.

## ANNOUNCEMENT

Severe acute respiratory syndrome coronavirus 2 (SARS-CoV-2) belongs to the genus *Betacoronavirus*, family *Coronaviridae*, and is the causative agent of the ongoing coronavirus disease 2019 (COVID-19) pandemic (1). Since the identification of the virus in late 2019, it has spread around the world, and the epidemiological picture is changing every day. We report here the coding-complete genome sequence of SARS-CoV-2 from a Pakistani patient who acquired the infection from Iran. This study was approved by the institutional review board (IRB) of the National University of Sciences and Technology, Islamabad, Pakistan (IRB reference number 03-2020-01/01). Informed consent was obtained from the patient whose information is included in this article.

A 30-year-old woman who had no history of comorbidities presented herself for a checkup at the local hospital in Gilgit, Pakistan, on 4 March 2020. She had previously traveled to Iran, arrived back in Gilgit, and in about 3 days developed a mild fever and sore throat. A nasopharyngeal swab sample was obtained from the patient in viral transport medium (VTM), and viral RNA was extracted using a High Pure viral RNA kit (Roche, Germany), according to the manufacturer’s recommendations. The sample tested positive for SARS-CoV-2 using the quantitative reverse transcriptase PCR (RT-qPCR) assay (2) on an Applied Biosystems ABI 7500 real-time PCR system, with a quantification cycle (*C_q_*) value of 14.

The SARs-CoV-2 genome was sequenced using the primer-walking method. A total of 48 sets of primers designed for heminested PCR and covering the whole-genome sequence of SARS-CoV-2, described in reference 3, were used. The PCR products were visualized on 1% agarose gel after electrophoresis and were Sanger sequenced using the ABI Prism sequencer. Individual amplicons were assembled using Geneious 11.1.5 with default parameters (4). A coding-complete genome sequence of 29,836 bp was obtained with a GC content of 38%. Available SARS-CoV-2 genome sequences were downloaded from GenBank using the BLASTN tool (5) with “2019-nCoV” as the query on 24 March 2020, and multiple sequence alignment was performed using the Geneious alignment algorithm. BLASTN and multiple sequence alignment yielded a query coverage of 99% to 100% of the complete genome sequences available in GenBank.

Mass scale genomic surveillance is needed to identify how many times the virus has reentered the population and which strains are currently present in Pakistan.

### Data availability.

The coding-complete genome sequence of this SARS-CoV-2 isolate has been deposited in GenBank under the accession number MT240479 (National Genomics Data Center of China accession number GWHACDD01000001). The version described in this paper is the first version, MT240479.1.
